# Feedback in formative OSCEs: comparison between direct observation and video-based formats

**DOI:** 10.3402/meo.v21.32160

**Published:** 2016-11-08

**Authors:** Noëlle Junod Perron, Martine Louis-Simonet, Bernard Cerutti, Eva Pfarrwaller, Johanna Sommer, Mathieu Nendaz

**Affiliations:** 1 Unit of Development and Research in Medical Education, Faculty of Medicine, University of Geneva, Geneva, Switzerland; 2 Division of Primary Care Medicine, Department of Community Medicine, Primary Care and Emergencies, Geneva University Hospitals, Geneva, Switzerland; 3 Service of General Internal Medicine, Department of General Internal Medicine, Rehabilitation and Geriatric Medicine, Geneva University Hospitals, Geneva, Switzerland; 4 Unit of Primary Care Teaching and Research, Faculty of Medicine, University of Geneva, Geneva, Switzerland

**Keywords:** formative, OSCEs, feedback, quality, video review, direct observation

## Abstract

**Introduction:**

Medical students at the Faculty of Medicine, University of Geneva, Switzerland, have the opportunity to practice clinical skills with simulated patients during formative sessions in preparation for clerkships. These sessions are given in two formats: 1) direct observation of an encounter followed by verbal feedback (direct feedback) and 2) subsequent review of the videotaped encounter by both student and supervisor (video-based feedback). The aim of the study was to evaluate whether content and process of feedback differed between both formats.

**Methods:**

In 2013, all second- and third-year medical students and clinical supervisors involved in formative sessions were asked to take part in the study. A sample of audiotaped feedback sessions involving supervisors who gave feedback in both formats were analyzed (content and process of the feedback) using a 21-item feedback scale.

**Results:**

Forty-eight audiotaped feedback sessions involving 12 supervisors were analyzed (2 direct and 2 video-based sessions per supervisor). When adjusted for the length of feedback, there were significant differences in terms of content and process between both formats; the number of communication skills and clinical reasoning items addressed were higher in the video-based format (11.29 vs. 7.71, *p=*0.002 and 3.71 vs. 2.04, *p=*0.010, respectively). Supervisors engaged students more actively during the video-based sessions than during direct feedback sessions (self-assessment: 4.00 vs. 3.17, *p=*0.007; active problem-solving: 3.92 vs. 3.42, *p=*0.009). Students made similar observations and tended to consider that the video feedback was more useful for improving some clinical skills.

**Conclusion:**

Video-based feedback facilitates discussion of clinical reasoning, communication, and professionalism issues while at the same time actively engaging students. Different time and conceptual frameworks may explain observed differences. The choice of feedback format should depend on the educational goal.

Formative objective structured clinical examinations (OSCEs) are considered to be useful opportunities for medical students to improve their clinical skills such as history taking, physical examination and communication skills. External feedback plays a crucial role in enhancing appropriate learning, correcting deficiencies, and monitoring students’ self-learning (1, 2) since self-assessment is not enough to ensure accurate identification of areas for improvement and to develop effective learning (3, 4).

Formative feedback can be given to students immediately after the clinical encounter or subsequently at a scheduled time. Immediate feedback after direct observation OSCE stations appears to improve subsequent student performance in a quick and durable way (5) and to enhance students’ self-assessment skills (6). Whereas students’ video review of their own performance does not lead to increased self-assessment accuracy (1, 2, 7, 8), video review with expert feedback appears to be more effective in terms of students’ satisfaction and performance and is superior to expert feedback alone (9, 10).

In 2013, we conducted a study among preclinical medical students to evaluate how well they perceived the feedback received from their supervisors in two different formative OSCEs: one based on direct observation and the other based on subsequent video review. Students rated the quality of feedback received during the video-based feedback session higher than feedback received during the direct observation (difference of 0.39 on a 1–5 Likert scale; *p<*0.001). This difference was independent of the clinical content of the OSCE and the characteristics of the supervisors involved in the feedback session (Junod Perron et al).

The aim of the study was to evaluate differences in the content and process of feedback in direct observation versus video-based OSCE formats.

## Methods

### Design, setting and participants

We conducted the study at the Faculty of Medicine, University of Geneva, Switzerland. The medical school offers a 6-year curriculum divided into three preclinical years (bachelor) and three clinical years (master) to 150 medical students per class. During the second and third preclinical years, medical students practice clinical skills during formative OSCEs in two different formats:

1. A 20-min interaction with a simulated patient followed by a 15-min feedback (direct observation) by a supervisor and 2–3 min of feedback by the simulated patient.
2. A 20-min videotaped interaction with a simulated patient, followed by immediate feedback by the simulated patient and a delayed 40-min feedback session by the supervisor 1 week later (including observation of the videotaped consultation), after the student has reviewed and analyzed his own performance (video-based feedback).

### Data collection

During the 2012–2013 academic year, all feedback sessions given to second- and third-year medical students during formative OSCEs were audiotaped. For each supervisor who had given both types of feedback, two feedback sessions given in the ‘direct observation’ format and two feedback sessions given in the ‘video-based format’ were randomly selected.

### Instrument

We adapted a previously developed instrument to assess the effectiveness of training on faculty feedback skills (11). The instrument, which assessed the way feedback is given, follows the structure of the MAAS-Global-Score, a well-known communication skills coding instrument, including both specific items, five global dimensions and a global rating item (12). Content validity was insured by an extensive review of the literature on effective feedback (5, 13–17). Construct validity was previously demonstrated in the ability of the instrument to detect improvements in faculty feedback skills (11). We added four items related to the content derived from the OSCE checklist used by supervisors and included three additional items specifically evaluating clinical reasoning and communication/professionalism.

### Outcome measures

#### Content of the feedback

Content analysis consisted of identifying and quantifying all themes mentioned and/or discussed during the feedback session. Themes were divided into seven categories (Table 1): global, history taking, physical examination, content of the explanation-end of encounter, communication, elaboration on clinical reasoning, and elaboration on communication and professionalism. ‘Elaboration’ referred to the number of times the supervisor elaborated in a directive or facilitative way on the importance of collecting elements of the history taking or physical examination in relation to the clinical reasoning process or underlined the importance of communication skills or attitudes for future practice during the feedback session.

**Table 1:** Content of the feedback given by the supervisors according to two types of formatives OSCE (12 supervisors, 48 audiotaped feedback)

Number of items addressed/discussed	Video-based, mean (SD)	Direct observation, mean (SD)	Delta (SE)	*p*	Adjusted to the length of the feedback, Delta (SE)	*p*
Global comments	0.50 (0.66)	0.63 (0.58)	−0.13 (0.19)	0.506	0.35 (0.35)	0.506
History taking	4.29 (3.21)	5.63 (3.21)	−1.33 (0.78)	0.097	−3.89 (1.38)	0.076
Physical exam	6.38 (2.78)	5.88 (2.51)	0.50 (0.72)	0.490	−1.12 (1.31)	0.476
Explanation – end of encounter	1.25 (1.19)	1.00 (1.06)	0.25 (0.32)	0.441	−0.66 (0.58)	0.416
Communication skills	11.29 (4.58)	7.71 (4.85)	3.58 (1.07)	0.002	1.05 (2.01)	0.002
Elaboration on clinical reasoning	3.71 (2.97)	2.04 (1.49)	1.67 (0.61)	0.010	−0.53 (1.10)	0.007
Elaboration on communication – professionalism	4.29 (1.68)	2.21 (1.38)	2.08 (0.40)	<0.001	1.12 (0.76)	<0.001

#### Process of the feedback

The process of feedback, focusing on the structure and the content of teaching skills used by the supervisors, was coded by N.J.P. and M.L.S. according to the 14 themes displayed in Table 1, using a 0–5 Likert scale (0 being absent and 5 optimal).

Interrater reliability, which was calculated on the basis of 10% of the audiotaped feedback sessions, was good (intraclass correlation coefficient=0.89).

In addition, all students who received feedbacks from supervisors involved in both formats were also asked to rate the utility and quality of the feedback immediately after the feedback session. The self-administrated questionnaire consisted of 14 items used in a previous study (Table 2) (Junod Perron et al).

**Table 2:** Process of the feedback given by the supervisors according to two types of formative OSCE (12 supervisors, 48 audiotaped feedback)

Feedback process^a^	Video-based, mean (SD)	Direct observation, mean (SD)	Delta (SE)	*p*	Adjusted to the length of the feedback, delta (SE)	*p*
The supervisor explored students’ learning needs	3.75 (1.07)	3.25 (1.19)	0.50 (0.29)	0.090	0.30 (0.57)	0.104
The supervisor stimulated students’ self-assessment	4.00 (1.32)	3.17 (1.17)	0.83 (0.29)	0.007	1.01 (0.57)	0.009
The feedback was descriptive	4.33 (0.96)	4.17 (0.82)	0.17 (0.21)	0.422	−0.08 (0.40)	0.433
The feedback was subjective	4.46 (1.18)	3.91 (1.35)	0.54 (0.28)	0.059	0.10 (0.54)	0.066
The feedback was balanced	3.92 (1.18)	4.21 (1.10)	−0.29 (0.28)	0.303	0.02 (0.51)	0.287
The supervisor took into account the student’s self-assessment	3.50 (1.59)	3.00 (1.35)	0.50 (0.35)	0.158	0.45 (0.68)	0.170
The supervisor stimulated the student to participate in the problem-solving process	3.92 (0.78)	3.42 (0.83)	0.50 (0.19)	0.012	−0.22 (0.34)	0.009
The supervisor used role-playing or hands-on practice to give students the opportunity to practice	1.75 (1.36)	1.46 (1.28)	0.29 (0.29)	0.321	−0.70 (0.53)	0.305
The supervisor checked the student’s understanding at the end of the feedback	3.79 (1.10)	3.25 (1.48)	0.54 (0.30)	0.083	0.35 (0.55)	0.069
Empathy	4.13 (0.74)	4.17 (1.13)	−0.04 (0.19)	0.825	−0.23 (0.37)	0.832
Pedagogical effectiveness	3.92 (0.72)	3.71 (0.91)	0.21 (0.17)	0.221	−0.17 (0.31)	0.216
Structure of the feedback	4.42 (0.83)	3.88 (0.95)	0.54 (0.16)	0.001	0.45 (0.30)	0.002
Verbal interaction	4.08 (0.58)	3.54 (1.06)	0.54 (0.19)	0.007	−0.08 (0.35)	0.007
Global evaluation	4.19 (0.55)	3.88 (0.78)	0.31 (0.12)	0.012	−0.03 (0.21)	0.009

a Likert scale 0–5: 0 being absent and 5 optimal.

The overall project was approved by the research ethics committee of the University Hospitals of Geneva. A complete review was waived by this committee.

### Analysis

Sociodemographic data were summarized by percentages, means, and standard deviation (SD). Feedback content data were expressed as the mean number of items addressed per session of feedback. Feedback process data were summarized by mean scores of Likert scale and SD. Comparisons of feedback scores (for both feedback content and process) were made with multivariate analysis of variance including a supervisor effect, and a feedback duration effect categorized in four groups (the quartiles were chosen as cut-off values). Differences in students’ perceptions regarding the quality of feedback between both formats were analyzed using the ANOVA test.

All analyses were run on R 2.15.3 (the R Foundation for Statistical Computing), and TIBCO Spotfire S+^®^ 8.1 for Windows (TIBCO Software Inc).

## Results

Twelve supervisors were involved in both direct observation and video-based feedback sessions. For each supervisor, two feedback sessions per format were randomly selected and analyzed (*n=*48).

Supervisors included seven women (7/12, 58%) and supervisors’ mean age was 45 years old (SD 10). Three worked in hospital-based general internal medicine, four in ambulatory general internal medicine, three as educationalists, and one as a hospital specialist. They had on average 18.2 years (SD 9.9) of clinical experience, had been clinical supervisors for an average of 9.4 years (SD 8.7), and were involved as supervisors in the formative OSCE for 4.8 years (SD 5.7).

Mean length of feedback was 26.2 min (SD 7.9) for the video-based sessions and 13.5 min (SD 4.0) for the direct observation sessions (*p<*0.001). Length of feedback in video-based sessions was measured as the actual time of verbal interaction, excluding video observation time.

During videotaped feedback sessions, supervisors addressed more often communication issues and elaborated more often on clinical reasoning and communication and professionalism issues than during direct observation sessions, independent of the length of feedback (Table 1).

Supervisors were also more learner centered during video-based feedback sessions (Table 2) and scored higher for several elements such as students’ self-assessment, student’s participation in the problem-solving process, and checking of students’ understanding, and transversal dimensions such as feedback structure and the amount of verbal interaction.

The 140 students who received feedback from supervisors involved in both formats perceived that in the video-based feedback, the supervisor made them more active by evaluating their learning needs, and by involving them more actively in the problem-solving. There was also some evidence that the video format was more effective than the direct observation format at improving their physical exam and communication skills (Table 3).

**Table 3:** Students’ perceptions of the quality of feedback according to the type of feedback format (12 supervisors and 140 students)

Quality of the feedback perceived by the students^a^	Video-based *n=*109, mean (SD)	Direct observation *n=*42, mean (SD)	*p*
The feedback session was useful	4.81 (0.46)	4.75 (0.57)	0.529
I improved my history taking skills	4.43 (0.74)	4.40 (0.75)	0.813
I improved my physical examination skills	4.51 (0.87)	4.20 (0.94)	0.063
I improved my communication skills	4.49 (0.71)	4.19 (0.90)	0.060
The supervisor was aware of what I needed to learn	4.90 (0.30)	4.82 (0.43)	0.269
The supervisor made me feel comfortable and confident	4.80 (0.46)	4.63 (0.67)	0.137
The supervisor asked me my learning needs	4.48 (0.96)	3.62 (1.55)	0.001
The supervisor asked me to evaluate what I did well	4.69 (0.52)	4.46 (0.91)	0.127
The supervisor asked me to evaluate what I could improve	4.93 (0.26)	4.77 (0.53)	0.067
The supervisor gave me balanced feedback (including both positive and less positive aspects)	4.93 (0.26)	4.69 (0.64)	0.022
The supervisor stimulated me to participate in the problem-solving process	4.56 (0.64)	4.00 (1.09)	0.003
The supervisor gave me precise and concrete suggestions for improvement	4.71 (0.64)	4.44 (0.90)	0.072
The supervisor provided me opportunities to practice parts of the history taking, physical exam, or the communication	3.18 (1.52)	2.77 (1.58)	0.165
The supervisor checked my understanding	3.58 (1.48)	3.37 (1.56)	0.473

a Likert scale 1–5: 1 completely disagree and 5 completely agree.

## Discussion

The results show that supervisors gave different feedback both in terms of content and process during video-based or direct observation formats, independent of the duration of feedback. In the video-based format, supervisors are more learner centered, address more communication and professionalism issues, and make more active links between history taking and physical examination and clinical reasoning. Students also perceived that the video format was more learner centered, and there was also some evidence that it was more effective at improving their communication and physical exam skills.

We found no literature about how the format in which formative OSCE takes place influences the way supervisors give feedback. In the field of simulation, the use of video-assisted debriefing is not superior to the use of non-video-assisted debriefing for the acquisition of technical and non-technical skills (18). However, regarding non-technical skills, as stated in two reviews, no study specifically analyzed the impact of the debriefing structure on learners’ perceptions and performance (19, 20).

Several factors may explain such differences of feedback formats. Direct observation and video-based formats use different frames of time. The first takes place immediately after the observed performance while the second is delayed. During the direct observation format, supervisors have a rather short and rigid time for feedback and teaching and may feel under pressure, preventing them to discuss and match their observations to students’ needs. The video-based format provides a longer and quieter time during which the supervisor may have more freedom and flexibility on how to organize time between observation, discussion, and teaching.

More importantly, supervisors may be encouraged to rely on different conceptual frameworks of learning according to the type of format. Argyris and Schön described two ways of learning inside an organization (21): *a single-loop learning* in which errors are detected and reflection is directed towards an immediate solution (problem-solving); *a double-loop learning* where errors are detected and reflection results more into questioning and even changing the overall framework than finding the more effective strategy. In the direct observation format, the fact that the supervisor spends more time talking and giving advice suggests a single-loop type of learning/teaching. Because the video-based format offers the student the opportunity to self-assess and reflect on his or her own performance, the supervisor may be more likely to act as a facilitator and to question students’ assumptions and frames of reference in a double-loop approach rather than providing immediate solutions. This results in more time for reflection, exchange, and elaboration of concepts and frames.

The reflective practice framework may also bring additional explanation to our observations (22, 23). In direct observation feedback, the student self-assessment and reflection ‘in action’ may be insufficient to provide the supervisor with elaborated materials to discuss. In the video-based feedback session, the students may have had sufficient time and opportunity to reflect on their actions, thus allowing for discussion about more elaborated self-reported materials during the feedback session.

Finally, because the video displays concrete and observable behaviors in a neutral way, it also avoids any source of misunderstanding between the student and the supervisor (9) and may stimulate the sharing of perceptions. This can be especially useful to teach dimensions such as communication and professionalism. Such issues often remain unaddressed during feedback not only because of supervisors’ lack of training or frames of reference (24) but also perhaps because of their reluctance and fear of being threatening or intrusive. This is of importance since several studies have shown a decline in empathy among students and residents during undergraduate and graduate medical education (25). Self-reflection during review of student videos linked to supervisor or peer feedback seems to be an effective and valued way to learn communication and professional attitudes (26, 27) and is more beneficial than traditional feedback on students’ communication skills (9). Video-based stimulated recall has also been shown to be useful to stimulate clinical reasoning or to facilitate development of shared cognition (28, 29). Thus, it seems that video-based feedback, by stimulating self-assessment, improves both self-awareness and skills (9, 30), especially if feedback is coupled to explicit performance expectations and benchmarks (8).

There are several limitations to our study. The sample of supervisors involved and the number of feedback sessions per supervisor analyzed were small. Second, the analysis of the feedback content, focused on counting the elements addressed during feedback, gives only a limited view of what was discussed during the session. Third, we only linked supervisors’ feedback to student perceptions of improvement and not to objective outcomes such as skill improvement or performance change. Finally, the fact that the study was conducted in only one medical school may limit the generalizability of our findings.

## Implications for practice

Our findings suggest that these two feedback formats may be applied differently according to the goals of the teaching session. For daily clinical supervision in busy clinics, short, immediate feedback following direct observation focusing on ‘single-loop’ reflection appears to be the most suitable format given time constraints. It is facilitated by the widespread use of the Mini-Clinical Evaluation Exercise (Mini-CEX), a work-based assessment used to evaluate a trainee’s clinical performance in real-life settings (31). Delayed and video-based feedback is more adapted for longer and less frequent sessions addressing issues such as professionalism, communication, and clinical reasoning where prior self-assessment, analysis and discussion of performance, and questioning of frames of reference may both increase students’ self-awareness and facilitate pedagogical diagnosis and remediation.

## References

[1] Hasnain M, Connell KJ, Downing SM, Olthoff A, Yudkowsky R (2004). Toward meaningful evaluation of clinical competence: the role of direct observation in clerkship ratings. Acad Med.

[2] Sandars J (2011). The e-learning site. Educ Prim Care.

[3] Eva KW, Regehr G (2005). Self-assessment in the health professions: a reformulation and research agenda. Acad Med.

[4] Ward M, Gruppen L, Regehr G (2002). Measuring self-assessment: current state of the art. Adv Health Sci Educ Theory Pract.

[5] Hattie J, Timperley H (2007). The power of feedback. Rev Educ Res.

[6] Reiter HI, Rosenfeld J, Nandagopal K, Eva KW (2004). Do clinical clerks provide candidates with adequate formative assessment during Objective Structured Clinical Examinations?. Adv Health Sci Educ Theory Pract.

[7] Backstein D, Agnidis Z, Regehr G, Reznick R (2004). The effectiveness of video feedback in the acquisition of orthopedic technical skills. Am J Surg.

[8] Srinivasan M, Hauer KE, Der-Martirosian C, Wilkes M, Gesundheit N (2007). Does feedback matter? Practice-based learning for medical students after a multi-institutional clinical performance examination. Med Educ.

[9] Hammoud MM, Morgan HK, Edwards ME, Lyon JA, White C (2012). Is video review of patient encounters an effective tool for medical student learning? A review of the literature. Adv Med Educ Pract.

[10] Ozcakar N, Mevsim V, Guldal D, Gunvar T, Yildirim E, Sisli Z (2009). Is the use of videotape recording superior to verbal feedback alone in the teaching of clinical skills?. BMC Public Health.

[11] Junod Perron N, Nendaz M, Louis-Simonet M, Sommer J, Gut A, Baroffio A (2013). Effectiveness of a training program in supervisors’ ability to provide feedback on residents’ communication skills. Adv Health Sci Educ Theory Pract.

[12] Van Thiel J, Kraan HF, Van Der Vleuten CP (1991). Reliability and feasibility of measuring medical interviewing skills: the revised Maastricht History-Taking and Advice Checklist. Med Educ.

[13] Cantillon P, Sargeant J (2008). Giving feedback in clinical settings. BMJ.

[14] Kluger NA, DeNisi A (1996). The effects of feedback interventions on performance: a historical review, a meta-analysis, and a preliminary feedback intervention theory. Psychol Bull.

[15] Hewson MG, Little ML (1998). Giving feedback in medical education: verification of recommended techniques. J Gen Intern Med.

[16] Kurtz S, Silverman J, Draper J (2005). Teaching and learning communication skills in medicine.

[17] Richardson BK (2004). Feedback. Acad Emerg Med.

[18] Cheng A, Eppich W, Grant V, Sherbino J, Zendejas B, Cook DA (2014). Debriefing for technology-enhanced simulation: a systematic review and meta-analysis. Med Educ.

[19] Garden AL, Le Fevre DM, Waddington HL, Weller JM (2015). Debriefing after simulation-based non-technical skill training in healthcare: a systematic review of effective practice. Anaesth Intensive Care.

[20] Sawyer T, Eppich W, Brett-Fleegler M, Grant V, Cheng A (2016). More than one way to debrief: a critical review of healthcare simulation debriefing methods. Simul Healthc.

[21] Argyris C, Schön D (1978). Organizational learning: a theory of action perspective.

[22] Mamede S, Schmidt HG (2004). The structure of reflective practice in medicine. Med Educ.

[23] Schön DA (1983). The reflective practitioner: how professionals think in action.

[24] Kogan JR, Conforti LN, Iobst WF, Holmboe ES (2014). Reconceptualizing variable rater assessments as both an educational and clinical care problem. Acad Med.

[25] Neumann M, Edelhauser F, Tauschel D, Fischer MR, Wirtz M, Woopen C (2011). Empathy decline and its reasons: a systematic review of studies with medical students and residents. Acad Med.

[26] Eeckhout T, Gerits M, Bouquillon D, Schoenmakers B (2016). Video training with peer feedback in real-time consultation: acceptability and feasibility in a general-practice setting. Postgrad Med J.

[27] Kalish R, Dawiskiba M, Sung YC, Blanco M (2011). Raising medical student awareness of compassionate care through reflection of annotated videotapes of clinical encounters. Educ Health (Abingdon).

[28] Balslev T, de Grave W, Muijtjens AM, Eika B, Scherpbier AJ (2009). The development of shared cognition in paediatric residents analysing a patient video versus a paper patient case. Adv Health Sci Educ Theory Pract.

[29] Nendaz MR, Gut AM, Perrier A, Reuille O, Louis-Simonet M, Junod AF (2004). Degree of concurrency among experts in data collection and diagnostic hypothesis generation during clinical encounters. Med Educ.

[30] Lane JL, Gottlieb RP (2004). Improving the interviewing and self-assessment skills of medical students: is it time to readopt videotaping as an educational tool?. Ambul Pediatr.

[31] Norcini JJ, Blank LL, Duffy FD, Fortna GS (2003). The mini-CEX: a method for assessing clinical skills. Ann Intern Med.

